# Assessment of Global Carbon Dioxide Concentration Using MODIS and GOSAT Data

**DOI:** 10.3390/s121216368

**Published:** 2012-11-26

**Authors:** Meng Guo, Xiufeng Wang, Jing Li, Kunpeng Yi, Guosheng Zhong, Hiroshi Tani

**Affiliations:** 1 Graduate School of Agriculture, Hokkaido University, Sapporo 060-8589, Japan; E-Mails: yikp@env.agr.hokudai.ac.jp (K.Y.); zhgs@env.agr.hokudai.ac.jp (G.Z.); 2 Research Faculty of Agriculture, Hokkaido University, Sapporo 060-8589, Japan; E-Mails: wang@env.agr.hokudai.ac.jp (X.W.); tani@env.agr.hokudai.ac.jp (H.T.); 3 Northeast Institute of Geography and Agroecology, Chinese Academy of Sciences, Changchun 130102, China; E-Mail: lijingsara@neigae.ac.cn

**Keywords:** MODIS, CO_2_ concentration, GOSAT TANSO, LST, NDVI/EVI, LAI/FPAR, GPP/NPP

## Abstract

Carbon dioxide (CO_2_) is the most important greenhouse gas (GHG) in the atmosphere and is the greatest contributor to global warming. CO_2_ concentration data are usually obtained from ground observation stations or from a small number of satellites. Because of the limited number of observations and the short time series of satellite data, it is difficult to monitor CO_2_ concentrations on regional or global scales for a long time. The use of the remote sensing data such as the Advanced Very High Resolution Radiometer (AVHRR) or Moderate Resolution Imaging Spectroradiometer (MODIS) data can overcome these problems, particularly in areas with low densities of CO_2_ concentration watch stations. A model based on temperature (MOD11C3), vegetation cover (MOD13C2 and MOD15A2) and productivity (MOD17A2) of MODIS (which we have named the TVP model) was developed in the current study to assess CO_2_ concentrations on a global scale. We assumed that CO_2_ concentration from the Thermal And Near infrared Sensor for carbon Observation (TANSO) aboard the Greenhouse gases Observing SATellite (GOSAT) are the true values and we used these values to check the TVP model accuracy. The results indicate that the accuracy of the TVP model is different in different continents: the greatest Pearson’s correlation coefficient (R^2^) was 0.75 in Eurasia (RMSE = 1.16) and South America (RMSE = 1.17); the lowest R^2^ was 0.57 in Australia (RMSE = 0.73). Compared with the TANSO-observed CO_2_ concentration (XCO_2_), we found that the accuracy throughout the World is between −2.56∼3.14 ppm. Potential sources of TVP model uncertainties were also analyzed and identified.

## 1. Introduction

Climate change is one of the great challenges of the 21st century [1]; the average surface temperature has increased by 0.74 °C over the past 100 years (1906∼2005). Climate exerts the dominant influence on the spatial distribution of the major vegetation types on a global scale. In turn, vegetation cover affects the climate via the alteration of the physical characteristics of the land surface, including albedo, roughness, water conductivity and atmospheric gas composition [2]. The increasing concentration of greenhouse gases (GHG) in the atmosphere has been verified as the most important cause of global warming, which is a major environmental concern and a prominent research topic [3–5]. According to the World Data Center for Greenhouse Gases [6], the average global carbon dioxide (CO_2_) concentration in 2010 was 389.0 ppm, which is 11.9 ppm greater than in 2004; this figure has increased 39% from the pre-industrial global level of 280.0 ppm. Observations of GHG in the atmosphere are important for predicting global climate change. However, it is difficult to observe the global variation of GHG because the direct sampling of gases, especially in the upper atmosphere, requires great effort and cost [7].

The increased atmospheric CO_2_ emissions from the burning of fossil fuels [8,9] and land-use changes [10–12] are such that terrestrial and marine environments are able to absorb approximately one-half to three-quarters of the emitted CO_2_[13,14]. To understand the relationship between land-cover changes and GHG concentration, great efforts have been made to measure the GHG emissions from forests and agricultural systems in recent years [11,15–17], and numerous data from field measurements and laboratory studies have been accumulated. However, global and regional GHG emissions estimates are still far from reliable because of the large spatial and temporal variations of the emissions records on which they are based.

There has been an intensive effort in recent years to measure and model the carbon cycles between the terrestrial biosphere and the atmosphere using a combination of modeling, remote sensing and observation data from eddy covariance flux towers. The eddy covariance technique offers an alternative for continuously and steadily assessing the long-term ecosystem carbon exchange. Furthermore, carbon budgets and the effects of environmental controls have been quantified with this technique for many forest types across the continent [11,17–21]. However, these carbon estimates from the eddy covariance technique only represent fluxes at the scale of the tower footprint, ranging from a hundred meters to several kilometers. To quantify the net exchange of CO_2_ over regions or continents, flux tower measurements need to be up-scaled to large areas [17]. In [22] the DeNitrification and DeComposition (DNDC) model, which focused on N_2_O and CO_2_ emissions, was developed. However, this model was mainly based on the cropping practices and soil conditions in the U.S. and China and cannot be used directly for the global carbon cycle [9,15]. An international network [23] has been formed to support such large-scale studies and provides standardized methods to develop “optical scaling” [24]. Today, more than 300 surface observation stations contribute to the WDCGG and World Meteorological Organization (WMO) by providing GHG data observed by flask sampling or *in situ*, measured worldwide at the Earth’s surface, from towers, onboard aircraft in the atmosphere and in oceanic environments [25,26]. Although ground-based measurements of GHG are highly accurate, they are sparse.

Launching satellites to collect GHG data can solve this issue quite well. At present, the Thermal And Near infrared Sensor for carbon Observation (TANSO) aboard the Greenhouse gases Observing SATellite (GOSAT), launched in 2009 by Japan, is the first instrument dedicated to record CO_2_ and methane (CH_4_) concentrations (denoted XCO_2_ and XCH_4_, in ppm. XCO_2_ and XCH_4_ are column-averaged dry air mole fractions of atmospheric CO_2_ and CH_4_) [8,27]. The SCanning Imaging Absorption spectroMeter for Atmospheric CHartographY (SCIAMACHY) aboard the ENVIronmental SATellite (ENVISAT), launched in 2002 and lost in 2012 by the European Space Agency (ESA), can also retrieve GHG concentrations, but it is not specially designed for GHG retrieval and is not highly accurate; the single-measurement precision is approximately 1∼2%, with an intermonth scatter of 2.3% [28,29]. Moreover, before 2002, the GHG concentrations could not be measured directly using the remote sensing technique.

To obtain CO_2_ concentration data using remote sensing data, [30] used CO_2_ fluxes with static chamber techniques and the normalized difference vegetation index (NDVI) data from a charged coupled device camera to analyze the relationship between NDVI and CO_2_ fluxes in arctic Alaska and found that the NDVI is not sufficient to estimate the carbon flux rate. Using remote sensing data and field data, [31] have developed models to map air-sea CO_2_ fluxes. To our knowledge, there is no model that can be used to retrieve CO_2_ concentrations from remote sensing data at a regional or global scale. Therefore, new approaches are critically needed to capture regional variations in CO_2_ concentration and to bridge a major gap between field and satellite observations [32,33].

In the present study, we assessed CO_2_ concentration using Moderate Resolution Imaging Spectroradiometer (MODIS) products globally from June 2009 to November 2011. The objectives were: (1) to examine the relationship between the MODIS-derived indices and TANSO XCO_2_; (2) to develop a monthly CO_2_ concentration model for Africa, Australia, Eurasia, North America and South America; and (3) to analyze the model uncertainties. This study explored the remote sensing potential for monitoring CO_2_ concentration at regional and global scales.

## 2. Study Area and Period

**Figure 1.**
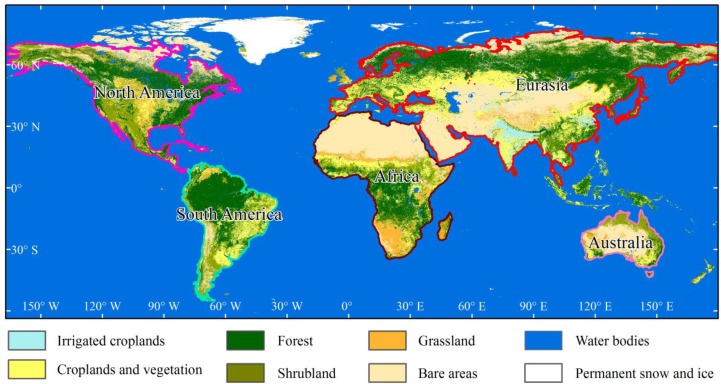
Study area. This is the 2009 global land-cover data that were downloaded from the ESA. The boundary lines of each continent are clearly shown. This figure also shows that in northern Africa, western Eurasia and most of Australia, the bare area rates are very high. Northern and southeastern North America, northern South America, central Africa and northern Eurasia have higher vegetation cover. Because of the smaller area, the study areas did not include some countries in Southeast Asia.

Because of the different climates, vegetation covers and human activity intensity in various regions of the World, it is difficult to derive CO_2_ concentrations using a single metric. To increase the derived accuracy of the CO_2_ concentration, we divided the World into five regions according to the continents: Africa, Australia, Eurasia, North America and South America (Figure 1). For each continent, we used different variables to assess CO_2_ concentrations. The annual NDVI in 2011 was 0.33, 0.36, 0.33, 0.41 and 0.59 for Africa, Australia, Eurasia, North America and South America, respectively. According to the ESA 2009 GlobCover data [34], which has a 300-m resolution, the bare area (the sum of sparse vegetation and bare area) rate in Africa, Australia, Eurasia, North America and South America is 36.77%, 41.79%, 31.20%, 10.9% and 15.3% respectively. The World’s largest desert, the Sahara, is located in northern Africa, leading to a lower vegetation cover in northern Africa. Australia is the driest continent in the World [35] and from Figure 1 we can see that the vegetation cover in all of Australia is very low. In Eurasia, the bare area (including the Arabian Desert and the Gobi Desert) is mainly distributed in western and central Asia. The Amazon River Basin in South America is mainly covered by evergreen tropical rainforests, which have a higher vegetation cover [36]. The total forest area in South America is approximately 9.5 × 10^6^ km^2^[37]. Moreover, South America is located near the Equator, and the higher annual temperatures and precipitation contribute to the higher NDVI in this region.

This study was conducted for data from June 2009 to November 2011 because the GOSAT TANSO data from April 2009 to November 2011 were available, but no such data exists for May 2009. The MODIS products for this time period were all available.

## 3. Data and Methods

### 3.1. MODIS Products Used in This Study

Optical remote sensing systems measure the surface reflectance, the fraction of solar energy that is reflected by the Earth’s surface. For a given wavelength, different land cover types may have different reflectance [16]. As a part of the National Aeronautics and Space Administration (NASA)-centered international Earth Observing System (EOS), two MODIS instruments (Terra and Aqua) have been launched (in 1999 and 2002, respectively) to provide information for global studies of atmospheric, land and ocean processes. The MODIS instrument strengths include its global coverage, high radiometric resolution and dynamic ranges and accurate calibration in the visible, near-infrared and thermal infrared bands [38].

MODIS, which is a key instrument aboard the Terra (AM) and Aqua (PM) satellites is one of the most reliable data sources on the global scale [21]. Terra and Aqua MODIS can view the entire Earth’s surface every 1 to 2 days, acquiring data in 36 spectral bands ranging in wavelength from 0.4 μm to 14.4 μm. The MODIS data have been used to generate science data products for more than eleven years and these science data products are available to the public free of charge. More than 10 types of products are retrieved from the 36 MODIS bands. In this study, we selected four product types, MOD11C3, MOD13C2, MOD15A2 and MOD17A2, to develop a CO_2_ concentration prediction model.

#### 3.1.1. MOD11C3 LST

The land surface temperature (LST) is retrieved from MODIS thermal bands data (bands 31 and 32) over the entire land surface of the Earth. The required surface emissivity in bands 31 and 32 are estimated from land cover types [39]. LST is derived from the energy balance at the soil-atmosphere interface and is therefore a crucial parameter for the environmental energy budget [39,40]. The atmospheric effects are corrected using the split-window algorithm, considering that the signal difference in the two TIR bands is caused by the atmospheric differential radiation absorption [41].

LST is a good indicator of the ground surface energy balance, as it is one of the key parameters in the physics of land-surface processes at regional and global scales [42]. LST is the result of the surface-atmosphere interactions and energy fluxes between the atmosphere and ground surface; therefore, it is widely used in climate, hydrologic, ecological and biogeochemical studies [11,41,43–46].

The MODIS LST accuracy is within 1 K in the range from 263 to 322 K under clear-sky conditions [47,48]. MODIS/Terra LST Monthly L3 Version 041 with a resolution of 0.05 degrees (MOD11C3) was used in this study.

#### 3.1.2. MOD13C2 NDVI/EVI

The Vegetation Index (VI) has become a common tool for assessing the different aspects of plant processes while simultaneously determining spatial variations in vegetation cover. The VI utilizes the differential absorption properties of leaves in different spectrum ranges to measure the amount and health of green biomass [30]. Two types of MODIS VIs, the NDVI and the Enhanced Vegetation Index (EVI), are globally produced over land at 1-km resolutions at 16-day compositing periods. Whereas the NDVI is chlorophyll sensitive, the EVI is more responsive to canopy structural variations, including canopy type, plant physiognomy and canopy architecture [49].

The NDVI has proven to be a valuable tool in researching large-scale changes in plant or ecosystem processes associated with global change. However, the actual range of the NDVI varies depending on the instrument used, background reflectance and canopy structure. Beyond a certain canopy density, increasing amounts of green biomass make little difference in the NDVI [50]. The EVI is more functional for Near Infrared (NIR) reflectance than for red absorption; therefore, it does not become saturated as rapidly as the NDVI in dense vegetation. Additionally, the EVI has been shown to be highly correlated with photosynthesis and plant transpiration [51]. The equations used in the NDVI and EVI are shown below (Equations 1 and 2):

(1) $$\mathrm{NDVI}=\frac{\rho_{\mathrm{nir}}-\rho_{\mathrm{red}}}{\rho_{\mathrm{nir}}+\rho_{\mathrm{red}}}$$

(2) $$\mathrm{EVI}=2.5\frac{\rho_{\mathrm{nir}}-\rho_{\mathrm{red}}}{\rho_{\mathrm{nir}}+\left(6\rho_{\mathrm{red}}-7.5\rho_{\mathrm{blue}}\right)+1}$$

where ρ_nir_, ρ_red_, and ρ_blue_ are the spectral reflectance in MODIS bands 2, 1 and 3, respectively [49,52].

The NDVI accuracy is within ±0.025, while that of the EVI is within ±0.015 and the accuracy of retrieving VI for a good quality day would be to within ±0.020 for NDVI and ±0.010 for EVI [53]. MODIS/Terra Vegetation Indices Monthly L3 Version 005 with a spatial resolution of 0.05 degrees (MOD13C2) were used in this study.

#### 3.1.3. MOD15A2 LAI/FPAR

Earth observations by the MODIS instruments on the Terra and Aqua satellites are being used to derive global vegetation properties such as the leaf area index (LAI) and absorbed fraction of photosynthetically active radiation (FPAR) at a 1-km pixel resolution and are updated once each 8-day period throughout each calendar year [24,47].

LAI is a biophysical variable influencing transpiration and photosynthesis at the plant canopy scale and carbon, water and energy dynamics at the regional scale [54]. The LAI is useful in measuring carbon flux because of its strong influence on canopy energy balance and rates of gas exchange [55]. It has been suggested, based primarily on theoretical studies, that canopy reflectance data provide more reliable indicators of canopy photosynthetically active radiation (PAR) interception and potential photosynthesis rates than do purely structural parameters such as the LAI and biomass [56].

The computation of the LAI from satellite data is based on a known relationship between the fractions of incident light transmitted through a canopy and the light extinction coefficient (k) (Equation 3):

(3) $$\mathrm{LAI}=\frac{-\text{ln}\left(1-\mathrm{FVC}\right)}{k\left(\theta \right)}$$

where the FVC (fractional vegetation cover) is defined as the vegetation-covered fraction of the ground viewed in the nadir direction and k(θ) is the light extinction coefficient for a solar zenith angle θ. For a detailed description of LAI, please see the paper by [57].

Converting LAI to FPAR uses a simple Beer’s Law approach, which enables the computation of FPAR as a function of LAI and the canopy light extinction coefficient (Equation 4):

(4) $$\mathrm{FPAR}=0.95\left(1-e^{-\mathrm{kLAI}}\right)$$

The value of the extinction coefficient k is 0.5 [58].

The accuracy of LAI is 0.66 LAI units RMSE when all biomes are taken in account. If broadleaf forests are excluded, the accuracy in all the other biomes is 0.5 LAI units RMSE. The accuracy is 0.12 FPAR units RMSE [53]. MODIS/Terra LAI/FPAR 8-Day L4 version 005 (MOD15A2) with a resolution of 1-km was used in this study.

#### 3.1.4. MOD17A2 GPP/NPP

The gross primary production (GPP) represents the capacity of the plants in an ecosystem to capture energy and carbon. The net primary production (NPP), the reduction of GPP after autotrophic respiration, is the net carbon stored as new plant material in an ecosystem, which supplies humans with various foods, fuels, fibers and construction materials [21]. Despite the strong theoretical and empirical relationship between surface reflectance and the FPAR, accurate estimates of vegetative productivity (GPP, NPP) depend strongly on the quality of the radiation inputs [59].

Global productivity can be estimated by combining remote sensing with carbon cycle processing [59]. The MODIS dataset provides the first operational, near-real-time calculation of the global GPP and NPP at a 250-m spatial resolution with daily coverage [10]. The MODIS GPP and NPP products have been validated as being able to capture spatial and temporal GPP and NPP patterns across various biomes and climate regimes, and the products are consistent with the ground flux tower-based GPP and field-observed NPP estimations [21].

The NPP product is designed to provide an accurate and regular measure of the production activity or growth of terrestrial vegetation. These products will have both theoretical and practical utility. The theoretical use is primarily to define the seasonally dynamic terrestrial surface CO_2_ balance for global carbon cycle studies [60,61]. Previous studies used tower and MODIS GPP/NPP data to model the carbon cycle in forests [11,17,50,62] and determined the relationships between GPP/NPP and LAI, NDVI and climate [10,62,63].

In addition, the NPP/GPP ratio (NG) [64] was found to be constant across a range of CO_2_ levels and temperature for herbaceous and woody plants. An assessment of the global patterns in the NG can enhance knowledge about features of the NG and benefit research on global change [21]. In the present study, we also attempt to use the GPP minus the NPP (GN) as a variable of the CO_2_ concentration model.

MODIS global annual estimates of GPP and NPP are within 10.4% and 9.0% [53]. MOD17A2 Monthly GPP/NPP with a resolution of 1 km, downloaded from the University of Montana [65], was used in the present work.

### 3.2. GOSAT TANSO Data

The GOSAT was launched on January 23, 2009 from Tanegashima Island, Japan, and is a joint project of the Japan Aerospace Exploration Agency (JAXA), the Ministry of the Environment of Japan (MOE) and the National Institute for Environmental Studies (NIES) of Japan. The primary purpose of the GOSAT project is to accurately estimate the emissions and absorptions of GHG on a subcontinental scale to assist environmental administrations in evaluating the carbon balance of land-based ecosystems and to provide assessments of regional emissions and absorptions [66]. The GOSAT main sensor (TANSO) is a nadir-looking Fourier Transform Spectrometer (FTS) with four spectral bands, from the visible to thermal infrared. GOSAT is the World’s first spacecraft designed to measure XCO_2_ and XCH_4_, the two major GHG concentrations, from SWIR bands with global coverage every three days [67]. GOSAT is a sun-synchronous orbit with a local overpass time of 13:00 [68,69]. It derives the CO_2_ and CH_4_ column amounts by observing ground-surface scattered solar spectra in the 1.6 and 2.0 μm regions, which contain suitable CO_2_ and CH_4_ absorption bands, and the satellite also measures vertical profiles higher than approximately 2 km by measuring thermal emission spectra from the atmosphere [8].

TANSO FTS SWIR Level 2 Version 02.00 and 02.11 (stored column abundances of CO_2_ and CH_4_ retrieved from the radiance spectra in bands 1 through 3 of FTS) data from June 2009 to November 2011 were used in this study. The relative accuracy of TANSO FTS Level 2 data is 0.3–1% (1∼4 ppm) for CO_2_[70]. The GOSAT FTS SWIR Level 2 data are point data and the footprint is a circle with 10.5 km diameter at nadir [71]; thus, we obtained the monthly CO_2_ concentration easily. Moreover, in this study, we assumed that the TANSO XCO_2_ were the true value without bias. TANSO XCO_2_ data were also used to check the accuracy of the modeled CO_2_ concentration from MODIS derived products.

### 3.3. Data Processes

MOD11C3, MOD13C2 and MOD15A2 were downloaded from the NASA homepage [74], and MOD17A2 was downloaded from the University of Montana [65]. Regarding the spatial resolution of these MODIS products, MOD11C3 and MOD13C3 have 0.05-degree spatial resolutions; MOD15A2 and MOD17A2 have 1-km resolutions. All of these products are sinusoidally projected, and all data are monthly except MOD15A2 (8-day). To use these data in one model, we must unify them into the same projection, the same spatial resolution and the same temporal resolution.

First, all data were reprojected and masked from a sinusoidal projection to the Geographic WGS84 projection as a 0.05-degree pixels using the MODIS Reprojection Tool [75]. Then, MOD15A2 LAI/FPAR was monthly stacked. We employed the maximum value composite (MVC) method that selected the highest pixel value to represent the composting period [17,49].

The spatial variability of GPP and NPP over the global is enormous, ranging from approximately 1,000 g C m^−2^ for evergreen tropical rain forests to less than 30 g C m^−2^ for deserts. For bare areas such as the Sahara Desert in North Africa and deserts in central-eastern Asia and western China, there are no calculated GPP data, and the values were defined as 0 in the present study.

For the TANSO CO_2_ data, we averaged all of the TANSO FTS Level 2 points in each month for each continent as the monthly value. From June 2009 to November 2011, there were 66,126 CO_2_ points in Africa, and the point numbers were 21,088, 37,651, 11,796 and 10,504 in Australia, Eurasia, North America and South America, respectively.

### 3.4. Linear Regression Model

The correlation coefficient (R), also called the Pearson correlation coefficient, provides an index of the degree of correlation between two datasets. In the present work, R^2^ was used to indicate the strength of the correlation between the modeled data set and the observations. The present work also employed the root mean square error (RMSE) to analyze the error between predictions and observations. The RMSE was used to evaluate the error of the predicted and the uncertainty relative to the TANSO-observed XCO_2_. The RMSE and R^2^, provide information on the regional performance and uncertainty of the modeled CO_2_ concentration.

## 4. Results

### 4.1. Seasonal Variations of the MODIS-Derived Indices on Each Continent

**Figure 2.**
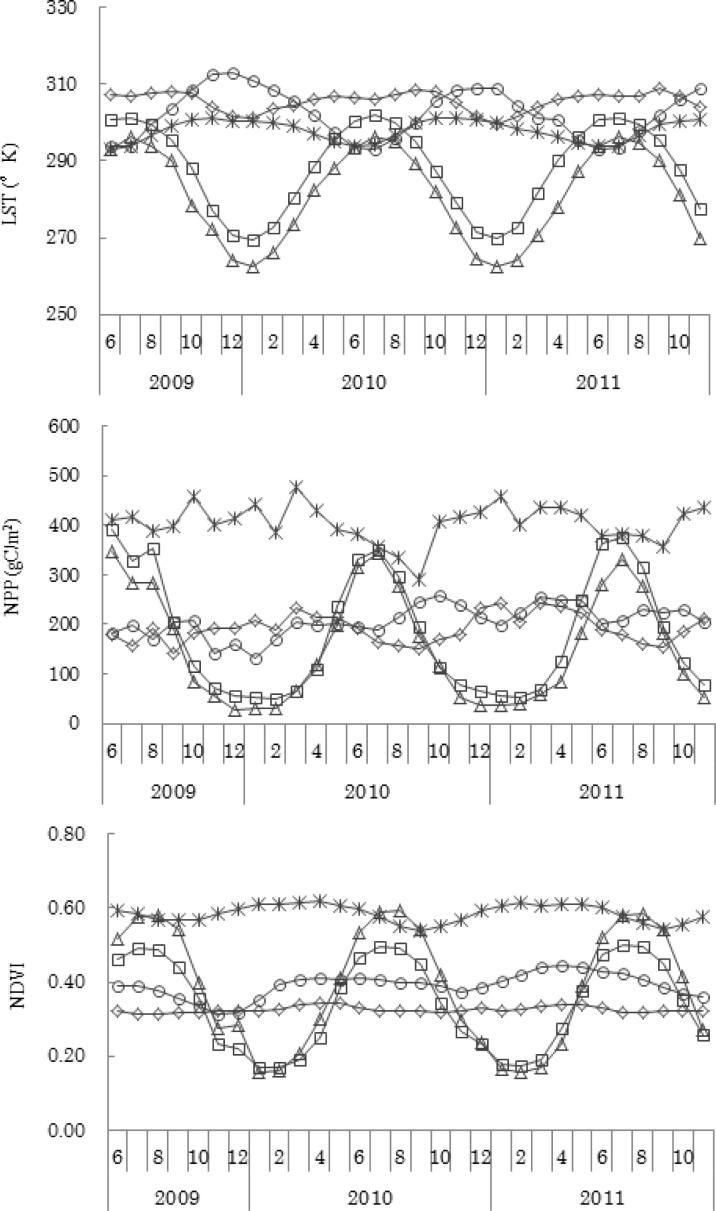

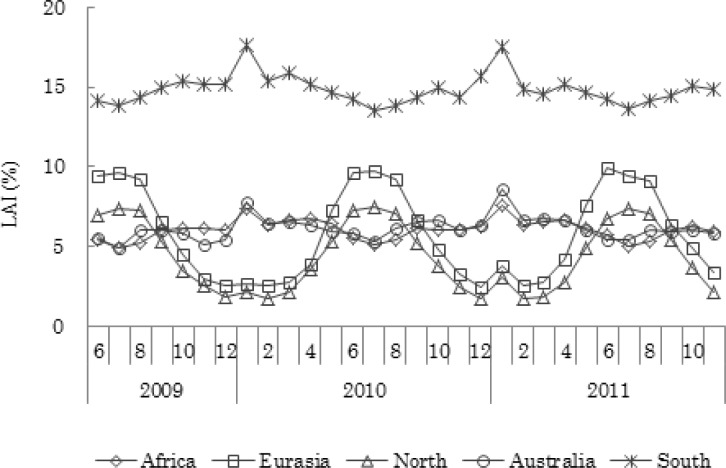
MODIS-derived indices trend of LST, NPP, NDVI and LAI from June 2009 to November 2011 in Africa, Australia, Eurasia, North America and South America.

MODIS-derived indices have different variations on different continents (Figure 2). In Eurasia and North America, all indices (LST, NPP, NDVI and LAI) change seasonally, with maximum values in summer (July or August) and minimum values in winter (January or February). The LST in Australia has the opposite trend from that found in Eurasia and North America, but the seasonal change is not as obvious. The fluctuation of NPP, NDVI and LAI are quite small in Africa, Australia and South America. South America always has the largest value of NPP, NDVI and LAI.

Eurasia and North America are located in Northern Hemisphere and the seasonal sensitivity of plant green-up and snow events are the main reasons of the changes in the indices. Australia is located in Southern Hemisphere, with a large percentage of bare areas leads to the small fluctuation of NPP, NDVI and LAI. The Amazon River Basin in South America has the highest vegetation cover in the world; thus, the NPP, NDVI and LAI are all higher in South America. The LST is closely associated with land-cover type, especially in bare areas [39], which is the main reason for higher value in of this parameter in Africa and Australia.

### 4.2. Correlation Analysis between TANSO XCO_2_ and MODIS-Derived Indices

The R^2^ and RMSE between TANSO XCO_2_ and MODIS-derived indices were analyzed across months on each continent. The following nine indices were considered in the analysis: LST (of MOD11C3), EVI and NDVI (of MOD13C2), LAI and FPAR (of MOD15A2), GPP, NPP, GN (GPP minus NPP) and NG (NPP/GPP) (of MOD17A2).

Different correlation coefficients between TANSO XCO_2_ and MODIS-based indices were found on different continents (Table 1). In Africa, the highest correlation coefficients were between XCO_2_ and NPP (R^2^ = 0.61 and RMSE = 1.21). Although the values between XCO_2_ and NDVI and LST were not very high (R^2^ = 0.39 and 0.24, RMSE = 1.53 and 1.70, respectively), but they meet the model indices select standard (see Section 4.3). In Australia, because the higher rate of bare areas can increase the reflectance of background, the highest correlation coefficient was between XCO_2_ and LST (R^2^ = 0.41, RMSE = 0.85). Although the bare areas in Eurasia are also extensive, the forests in Siberia and southern China increase the correlation between XCO_2_ and NDVI and EVI (R^2^ = 0.44 and 0.42, RMSE = 1.73 and 1.76, respectively). In North America, the correlation coefficients between XCO_2_ and NDVI reached R^2^ = 0.65 (RMSE = 1.15) and the lowest values that were selected for the TVP model is R^2^ = 0.49 (between XCO_2_ and LST and NDVI). In South America, the highest correlation coefficients were between XCO_2_ and LST (R^2^ = 0.64, RMSE = 1.39). The index of NG (R^2^ = 0.19, RMSE = 2.09) were also selected for the model build.

The different values indicate that CO_2_ concentration was influenced by different factors associated with different climate and land-cover types [2]. Table 1 also shows that LST was important in all five continents, which indicates that LST is an important factor in CO_2_ concentration research. With regard to the selected indices in the five continents, only Australia had an average RMSE lower than 1; Eurasia had the highest averaged RMSE (1.87).

### 4.3. Assessment of Modeled CO_2_ Concentration

In this study, we used the MODIS products of temperature (MOD11C3), vegetation cover (MOD13C2 and MOD15A2) and productivity (MOD17A2) as variables to measure land surface CO_2_ concentration; thus, we named the model TVP. To select the indices of the TVP model, first, we calculated the correlation coefficient between each of these indices and the TANSO XCO_2_ on each continent (Table 1). Then, we selected indices according to higher R^2^, lower RMSE and significance value (P); if P was less than 0.05 (significance greater than 95%) and the index increased the correlation coefficient in the multivariate linear regression of the TVP model, then the index was selected. Finally, we selected seven indices for Eurasia and North America, six indices for South America and five indices for Africa and Australia. The results are shown in Table 1. EVI and NDVI, GPP and NPP and LAI and FPAR are pairs of indices that measure the same parameter and can, in some cases, be substituted for one another. Researchers found that a small number of indices is not sufficient to provide an accurate estimate of CO_2_ flux [30]; thus, we also tested the use of a single index for a given parameter (e.g., NPP but not GPP in Africa and NDVI but not EVI in Eurasia), but we found that the adjusted R^2^ value was lower than that of the TVP model. At last, the index number that been selected for TVP model is 5 in Africa and Australia, 7 in Eurasia and North America and 6 in South America. After analyzing the R^2^ and RMSE between MODIS products and the TANSO XCO_2_, we employed MODIS-derived indices that were selected for each continent as independent variables and TANSO XCO_2_ as dependent variables of multivariate linear regression in Microsoft Office 2010 to model the CO_2_ concentration on each continent. The TVP models for each continent are described below:

Africa:
  (5) $${\text{CO}}_{2}=396.06-0.06\times \text{LST}+89.49\times \text{NDVI}-0.13\times \text{GPP}+0.23\times \text{NPP}-49.18\times \text{NG}$$
Australia:
  (6) $${\text{CO}}_{2}=362.73+0.07\times \text{LST}+29.02\times \text{NDVI}+0.03\times \text{NPP}-23.90\times \text{NG}-0.07\times \text{GN}$$
Eurasia:
  (7) $$\begin{array}{c} \begin{array}{c} {\text{CO}}_{2}=277.93+0.40\times \text{LST}+95.04\times \text{EVI}-64.32\times \text{NDVI}-0.89\times \text{FPAR}+2.73\times \\ \text{LAI}-0.03\times \text{GPP}-0.004\times \text{GN} \end{array} \end{array}$$
North America:
  (8) $$\begin{array}{c} {\text{CO}}_{2}=321.04+0.23\times \text{LST}+48.44\times \text{EVI}-27.06\times \text{NDVI}-0.52\times \text{FPAR}+1.32\times \\ \text{LAI}-0.03\times \text{GPP}-0.01\times \text{GN} \end{array}$$
South America:
  (9) $$\begin{array}{c} {\text{CO}}_{2}=634.59-0.49\times \text{LST}-9.49\times \text{EVI}-0.66\times \text{LAI}+0.11\times \text{GPP}-166.04\times \text{NG}-\\ 0.27\times \text{GN} \end{array}$$

**Figure 3.**
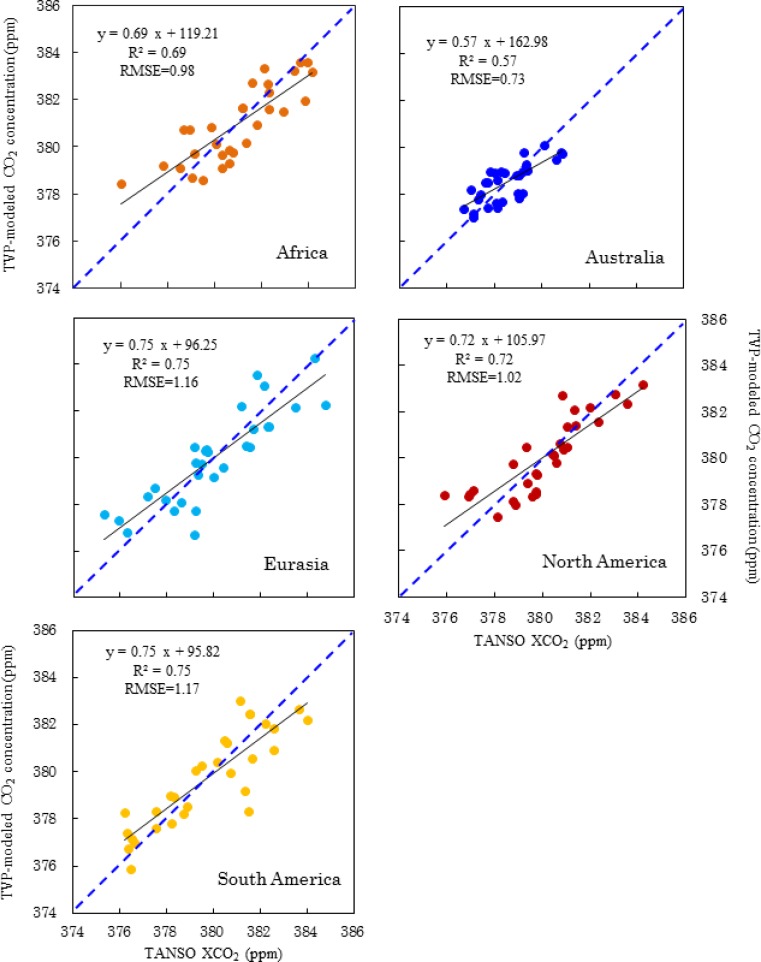
Comparison of TVP modeled with TANSO-observed CO_2_ concentration in Africa ( 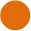), Australia ( 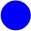), Eurasia ( 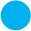), North America ( 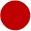) and South America ( 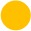). The dotted blue line is the 1:1 line between TVP derived and TANSO observed XCO_2_. The black line is the regression line.

TVP models were run on a monthly time scale using the data of LST, EVI, NDVI, LAI, FPAR, GPP, NPP, GN and NG to predict CO_2_ concentrations for the five continents. The TVP model performance was then evaluated using scatter plots (Figure 3) of the predicted versus TANSO-observed CO_2_ concentrations from June 2009 to November 2011.

Figure 3 shows that the correlation coefficient was not very high (R^2^ = 0.57, RMSE = 0.73) in Australia. The TVP model provided better estimates of CO_2_ concentration for Eurasia and South America (R^2^ = 0.75 for both, RMSE = 1.16 and 1.17, respectively). The validation points of the TVP-modeled and TANSO-observed XCO_2_ of Africa and North America were distributed closely around the 1:1 line (RMSE = 0.98 and 1.02, respectively). Satisfactory correlation coefficients were found in both Africa and North America (R^2^ = 0.69 and 0.72, respectively). Thus, the results were very encouraging on all five continents. Notably, the higher R^2^ values for the TVP-modeled CO_2_ concentration in North America and South America do not necessarily indicate that the accuracy was higher for these continents. We found that in North and South America, the TANSO CO_2_ points alone were inadequate (11,796 and 10,504, respectively) because the TANSO sensor is only able collect points under a clear sky (without clouds) [28]. As mentioned in Section 3.2 that the accuracy of TANSO XCO_2_ is about 0.3∼1% and it must increasing the uncertainty of the correlations between predicted and observed CO_2_ concentrations if we presume TANSO XCO_2_ as true value. Moreover, in different continents the bias of TANSO XCO_2_ is different [28,72,73,76], which can also increase the uncertainly of Figure 3. These uncertainties have not necessarily weakened this study, in terms of achieving the targeted objectives.

Figure 3 also shows that the slope of predicted versus observed CO_2_ concentration was significantly lower than the 1:1 line, suggesting that the TVP algorithm somewhat underestimates high CO_2_ concentration and overestimates low CO_2_ concentration. However, the over/underestimation of extreme values had only a small impact on the predicted value, with the largest RMSE at 1.17 in South America and the lowest at 0.73 in Australia.

## 5. Discussion

### 5.1. Seasonal Variation and the Differences between TVP-Modeled and TANSO-Observed CO_2_ Concentration

**Figure 4.**
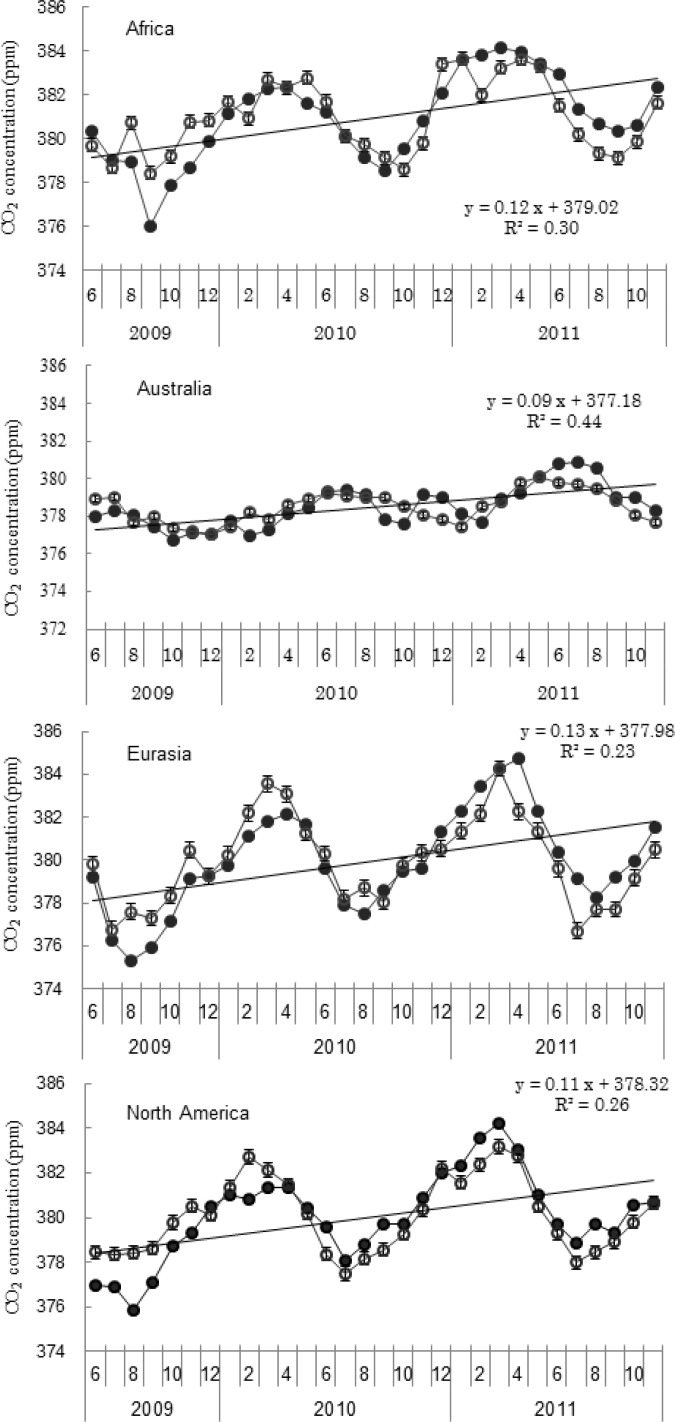

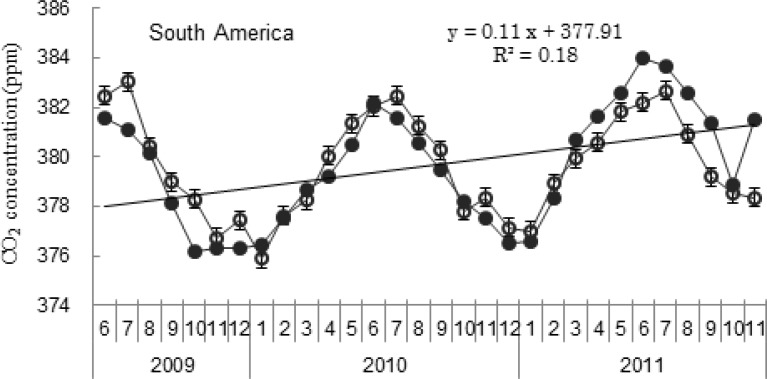
The seasonal variations of CO_2_ concentrations on each continent and the differences of TVP-estimated CO_2_ concentration compared with the TANSO-observed values. Hollow points are the TVP-modeled CO_2_ concentration and solid points are the TANSO-observed values. The lines are the trend line of TANSO XCO_2_.

Figure 4 shows the seasonal variation of CO_2_ concentration and the differences between TVP-modeled and TANSO-observed CO_2_ concentration. The TANSO XCO_2_ time series showed a more pronounced seasonal variation and the extreme values appeared in different months on different continents. In Africa, the observed CO_2_ concentration ranged between a maximum of 384.13 ppm and a minimum of 376.00 ppm between June 2009 and November 2011. The maximum and minimum appeared in March and September, respectively. The CO_2_ concentration estimated by TVP model were overestimated before September 2010 and underestimated in 2011. The largest difference between the modeled and observed CO_2_ concentration was −2.43 ppm, which occurred in September 2009. In Australia and South America, the maximum value of CO_2_ concentration was usually in June or July and the minimum value appeared around December. Moreover, Figure 4 shows that the fluctuation of CO_2_ concentration from June 2009 to December 2011 was very mild in Australia, where the difference between minimum and maximum value of TANSO XCO_2_ was 4.17 ppm. This low fluctuation mainly results from the land-cover types in Australia, where bare areas covered 41.79%. The amount of CO_2_ sequestered by desert soils is lower than by other soils [77]. Lack of plant photosynthetic is the main reason for the mildly seasonal change of CO_2_ concentrations. The largest differences between TVP-modeled and TANSO-observed CO_2_ concentration was 1.24 ppm in Australia and the average value is 0.62 and 0.95 ppm in Australia and South America, respectively. In Eurasia and North America, the minimum values of CO_2_ concentration appeared in August and the maximum values appeared in April or March. The difference between minimum and maximum value of TANSO XCO_2_ was 9.46 and 8.39 ppm in Eurasia and North America, respectively. In Eurasia, overestimating of CO_2_ concentration by TVP model was found from June 2009 to April 2010 and underestimating appeared after December 2010. In North America, the overestimating happened from June 2009 to April 2010 (except December 2009) and from then on underestimating happened (except December 2010). The average difference between TANSO-observed and TVP-modeled CO_2_ concentration was 0.97 and 0.84 ppm in Eurasia and North America, respectively. In Africa, the maximum overestimating of TVP model was 2.43 ppm in September 2009 and the maximum underestimating value was 1.86 ppm in February 2011.

Figure 4 also shows that in the study time scale, TANSO XCO_2_ clearly increased in all the study areas. The maximum slope was in Eurasia (slope = 0.13) and the minimum slope was in Australia (slope = 0.09).

### 5.2. Uncertainties and the Significances of the TVP Model

Some studies have used ground-based data to validate the CO_2_ concentration estimated by TANSO and SCIAMACHY and found different results in different regions. TANSO XCO_2_ with six ground-based Total Carbon Column Observing Network [78] sites around the world have been compared and found that the average bias of TANSO XCO_2_ was 2.80 ppm [28]. Researchers also found that the computed errors of TANSO XCO_2_ varied between 1.20 and 3.20 ppm [72]. With regard to the accuracy of SCIAMACHY CO_2_ concentration, some researchers found that the global single-measurement precision amounted to 2.50 ppm [29,79]. In the present study, we used TANSO XCO_2_ as the observed value to check the accuracy of TVP model and found that the accuracy in the study area was between −2.56∼3.14 ppm. Australia exhibited the lowest difference, at −1.24∼1.19 ppm, and the largest difference was in South America, at −2.09∼3.14 ppm. It indicates that the accuracy of TVP model is similar with the SCIAMACHY and TANSO.

Despite the encouraging performance of the TVP model in estimating CO_2_ concentration from MODIS products, these estimates still contain significant uncertainties and are a long way from reliably estimating CO_2_ concentration from remote sensing data. Although random error is usually considered less serious than uncertainties [31], there can be many sources of error that create a high overall uncertainty. The CO_2_ exchange between terrestrial ecosystems and the atmosphere has been mainly dominated by three factors: climate, land-cover type and human activities [4,80–82]. With regard to the TVP model in the present study, there are several sources of uncertainty associated with the CO_2_ concentration estimates: uncertainly in the TANSO accuracy, uncertainty in land cover, uncertainty in the input data and uncertainty regarding the extent and nature of climate change in different regions. Thus, there remains a great deal of research is still required to ensure the accuracy of CO_2_ concentration estimated based on remote sensing data.

Although the TVP model itself has some shortcomings but it is a meaningful explore in CO_2_ calculate using remote sensing data. The same as used MODIS, we can also use NOAA AVHRR data combine with ground stations data to derive CO_2_ and CH_4_ concentrations at regional or global scale. Using the TVP model we can derive global CO_2_ concentration from 1981 to 2002 when there was no GHG satellite. In fact, we can also use TVP model to identify the spatial distribution of GHG concentrations which is also an important issue nowadays. Moreover, in the future, with the launching of other GHG observe satellites, e.g., Orbiting Carbon Observatory 2 (OCO-2), Carbon Monitoring Satellite (CarbonSat) and GOSAT-2, the accuracy of TVP model will increasing.

## 6. Summary and Conclusions

TANSO, onboard the GOSAT satellite launched on January 23, 2009, is the first and only orbiting greenhouse gas observing satellite. Although SCIAMACHY, onboard ENVISAT, has also been able to measure greenhouse gas concentrations since 2002, it is not specifically designed for studying GHG, and the data require calibration. Approximately 300 stations are far apart from accurately global GHG concentration measure. The present study demonstrates that MODIS derived indices have great potential for CO_2_ concentration measurement on regional as well as global scales.

The assessment of CO_2_ concentration from remote sensing data has been conceived and attempted in the present work. First, we examined the relationships between the MODIS derived indices and CO_2_ concentration on each continent and found that CO_2_ concentration is dominated by different factors in different continents. LST, NDVI and GPP are the main factors that affect the CO_2_ concentration and their relative contributions are different in different areas. Second, we developed a TVP model for CO_2_ concentration measurement based on the relationships between the MODIS derived indices and CO_2_ concentration and got satisfactory results. The accuracy of TVP model is different on different continents: the highest correlation coefficient between the modeled and observed value is in Eurasia and South America (R^2^ = 0.75, RMSE = 1.16 and 1.17, respectively); the lowest value is found in Australia (RMSE = 0.73). Compared with the TANSO XCO_2_, we found that the accuracy across the study area is between −2.56∼3.14 ppm. From the results we also found that the TVP model underestimates high CO_2_ concentration and overestimates low values.

Increasing the accuracy of CO_2_ concentration that derived from remote sensing data remains beyond current capabilities. The TVP model that proposed in this study also contains some uncertainties (described in Section 5.3). With the releasing of CO_2_ concentration data by GOSAT project, validated data at different time scales should be used in future studies.

## Figures and Tables

**Table 1.** Pearson’s correlation coefficient (R^2^) and the root mean square error (RMSE) between TANSO XCO_2_ and MODIS-derived indices. Gray masked indices are selected for the TVP model.

		LST	EVI	GPP	NPP	NG	GN	NDVI	FPAR	LAI
	
Africa	R^2^	0.24	0.09	0.47	0.61	0.40	0.01	0.39	0.23	0.19
	P	0.007	0.104	0.000	0.000	0.000	0.576	0.000	0.008	0.016
	RMSE	1.70	1.86	1.42	1.21	1.50	1.94	1.53	1.72	1.75

		LST	EVI	GPP	NPP	NG	GN	NDVI	FPAR	LAI
	
Eurasian	R^2^	0.22	0.42	0.31	0.26	0.01	0.35	0.44	0.35	0.29
	P	0.009	0.000	0.002	0.004	0.543	0.001	0.000	0.001	0.002
	RMSE	2.04	1.76	1.92	1.98	2.29	1.87	1.73	1.86	1.94

		LST	EVI	GPP	NPP	NG	GN	NDVI	FPAR	LAI
	
North America	R^2^	0.49	0.59	0.51	0.49	0.00	0.45	0.65	0.59	0.55
	P	0.000	0.000	0.000	0.000	0.796	0.000	0.000	0.000	0.000
	RMSE	1.37	1.23	1.35	1.37	1.92	1.42	1.15	1.23	1.30

		LST	EVI	GPP	NPP	NG	GN	NDVI	FPAR	LAI
	
Oceania	R^2^	0.41	0.13	0.00	0.16	0.35	0.20	0.38	0.11	0.04
	P	0.00	0.05	0.99	0.03	0.00	0.01	0.00	0.08	0.28
	RMSE	0.85	1.04	1.11	1.02	0.90	1.00	0.88	1.05	1.09

		LST	EVI	GPP	NPP	NG	GN	NDVI	FPAR	LAI
	
South America	R^2^	0.64	0.30	0.42	0.13	0.19	0.61	0.00	0.11	0.46
	P	0.000	0.002	0.000	0.048	0.016	0.000	0.777	0.080	0.000
	RMSE	1.39	1.94	1.77	2.16	2.09	1.46	2.32	2.19	1.71
